# Inequalities in older LGBT people's health and care needs in the United Kingdom: a systematic scoping review

**DOI:** 10.1017/S0144686X19001326

**Published:** 2021-03

**Authors:** Dylan Kneale, Josie Henley, James Thomas, Robert French

**Affiliations:** 1 EPPI-Centre, UCL Institute of Education, University College London, London, UK; 2 Centre for Trials Research, College of Biomedical and Life Sciences, Cardiff University, Cardiff, UK; 3 School of Medicine, Cardiff University, Cardiff, UK

**Keywords:** lesbian, gay, bisexual, transgender, health, social care, inequalities, older people

## Abstract

The hostile environment that older lesbian, gay, bisexual and transgender (LGBT) people faced at younger ages in the United Kingdom (UK) may have a lasting negative impact on their health. This systematic scoping review adds to the current knowledge base through comprehensively synthesising evidence on what is known about the extent and nature of health and care inequalities, as well as highlighting gaps in the evidence which point the way towards future research priorities. We searched four databases, undertook manual searching, and included studies which presented empirical findings on LGBT people aged 50+ in the UK and their physical and mental health or social care status. From a total of 5,738 records, 48 papers from 42 studies were eligible and included for data extraction. The synthesis finds that inequities exist across physical and mental health, as well as in social care, exposure to violence and loneliness. Social care environments appeared as a focal point for inequities and formal care environments severely compromised the identity and relationships that older LGBT people developed over their lifecourse. Conversely, the literature demonstrated how some older LGBT people successfully negotiated age-related transitions, *e.g.* emphasising the important role of LGBT-focused social groups in offsetting social isolation and loneliness. While there exist clear policy implications around the requirement for formal care environments to change to accommodate an increasingly diverse older population, there is also a need to explore how to support older LGBT people to maintain their independence for longer, reducing the need for formal care.

## Introduction

### LGBT people's health and health inequalities

For older lesbian, gay, bisexual and transgender (LGBT) people in the United Kingdom (UK), the legislative landscape has changed beyond recognition during their lifetime. Most people who could conventionally be regarded as being on an ‘ageing trajectory’ (aged 50+), if not ‘older’ *per se*, were born at a time where male homosexuality was, in effect, criminalised, and where social and legislative conditions permitted discrimination across a wide spectrum of domains for men and women from sexual minorities. Even after advances in the 1960s and 1970s such as the decriminalisation of same-sex acts between men in 1967, the legislative and social landscape remained hostile. During the 1980s, when the last of the baby-boom generation were experiencing transitions to adulthood, the onset of the HIV/AIDS epidemic had devastating impact on the health, wellbeing and social networks of LGBT people. Since the 1990s, the legislative landscape in the UK has continued to become more permissive, so that LGBT people can access similar rights and treatment to heterosexual people across a range of domains. Compared to the legislative landscape, the pace of change across the social landscape has been more gradual, with a sizeable minority of the UK public continuing to regard same-sex relationships as ‘always or mostly wrong’ (Park and Rhead, 2013). Meanwhile, the legislative and social landscape for transgender people in the UK has lagged further behind, with the right to change the gender assigned at birth only being recognised in UK law in 2004, despite the European Court of Human Rights recognising this as a fundamental human right 15 years earlier.

Existing evidence is suggestive that LGBT people are at risk of poorer health outcomes across a range of domains (Meyer and Northridge, 2007; Blosnich *et al*., 2014); this is theorised to be an artefact of the negative social climate that LGBT people have experienced (Frost *et al*., 2015). However, the evidence does not necessarily indicate systematic inequality across all health outcomes, or among all people included within the LGBT acronym, and there exist other questions around the robustness and generalisability of the findings across the UK and across the generations. The extent to which a drive towards equality in treatment in health and care services can inadvertently overlook any specialist needs that LGBT people may have is also uncertain.

The literature exploring the lives of older LGBT people in the UK has, even in the recent past, been characterised as small and underdeveloped (Musingarimi, 2008*a*, 2008*b*; Potter *et al*., 2011), and evidence that could help decision-makers and practitioners working in health and care settings to tailor their services has been difficult to identify and synthesise. Notably, empirical evidence on transgender health needs in later life, or even across other lifecourse stages, has been characterised as almost entirely absent, despite the unique stresses and challenges transgender people face later in life (Williams and Freeman, 2007).

Earlier (non-systematic) reviews suggested that older LGBT people are at risk of poor health and care outcomes (Musingarimi, 2008*a*, 2008*b*; Potter *et al*., 2011), although the evidence included within these reviews is (a) piecemeal and identified ‘serendipitously’, (b) based in part on studies conducted beyond the UK with different health and care systems and contexts for LGBT people; and (c) may have been superseded by evidence published since. Nearly a decade after the publication of these previous reviews, we are theoretically in a better position to plug evidence gaps previously identified, with a greater number of surveys having collected information on the lives of LGBT people in the UK, including those co-ordinated by the Office for National Statistics (Taylor, 2008; Joloza *et al*., 2010), as well as a broader emergent interest in the lives of older LGBT people and health among care providers (Page *et al*., 2016; Willis *et al*., 2017; Hafford-Letchfield *et al*., 2018) and researchers (Wellcome Trust, 2017; King *et al*., 2018).

This systematic scoping review explores how the health and care needs of older LGBT people differ and represent inequalities between older LGBT and non-LGBT people in the UK, and follows a protocol predating this review (Kneale *et al*., 2018). This review seeks to contribute to the knowledge base through both synthesising evidence on what is known about the extent and nature of health and care inequalities, as well as highlighting gaps in the evidence which can help to direct future research priorities.

### Exploring fuzzy concepts and health inequalities among older LGBT people

This review deals with three fuzzy concepts – older people, LGBT people and health inequalities – that become ‘fuzzier’ when considered in relation to each other. Beginning with the concept of older people, many studies have conventionally used the State Pension Age, to delineate people as being ‘older’. For LGBT people, and gay men in particular, a theory of ‘accelerated ageing’ contends that older age is perceived as being reached at a much younger point than for heterosexual men (Schope, 2005), and some studies of ‘older’ gay men follow suit, imposing relatively young thresholds on studies of older gay men (Hughes and Deutsch, 2010). In this study, we also impose a relatively young threshold for age of 50 years, reflecting both the subjectivity around the interpretation of older age, as well as to capture evidence on ‘ageing trajectories’.

Our second fuzzy concept is around our definition of LGBT itself and particularly its ascription to older people. King and Cronin (2013) describe the tension in applying an ‘LGBT’ identity to older people, where older people have historically been represented (and understood) as being sexually inactive. Another element is that this identity is viewed as fixed, in opposition to the fluidity demonstrated by queer theorists (King and Cronin, 2013), and the evidence gathered empirically from older people by researchers (Knocker, 2012). For older people in particular, whose identity was developed in periods of substantial hostility, this may lead to underreporting of sexual orientation (Joloza *et al*., 2010) when compared to reports of same-sex experience (Kneale and French, 2018). Geary *et al*. (2018) illustrate this discrepancy in their study, finding that only 1 per cent of men aged 65–74 identified as LGBT with over three times as many reporting same-sex experiences (3.4%); a similar, albeit less marked, pattern was observed for women. Nevertheless, those who have same-sex experiences but who do not identify as LGBT are found to experience similar health disparities compared to those who exclusively have opposite-sex experiences (Mercer *et al*., 2016), suggesting health inequalities extend beyond LGBT identity alone. This review synthesises evidence on LGBT people based on sexual identity, sexual attraction and sexual experience.

Our third fuzzy concept is around ‘health inequalities’. Health inequalities are systematic differences in the health status between two groups. When health inequalities cannot be explained by naturally occurring differences, but are instead driven by social injustice, they are regarded as health inequities (Kawachi *et al*., 2002). Measures of health inequality such as the concentration index and the index of inequality provide summaries of the severity of health inequalities, particularly those that appear to be driven by socio-economic inequalities (Wagstaff *et al*., 1991). However, health inequalities represent a fuzzy concept in this review, given that the focus on health extends beyond the ‘crisp’ concepts usually measured in studies of health inequalities, such as life expectancy, to include wider health-related domains pertinent to older people, such as care. A rigid focus on health ‘inequalities’ could also lead us to assume that the health needs of older LGBT people mirror those of heterosexual people, and to overlook specific needs of older LGBT people. This review adopts a ‘fuzzy’ perspective on health inequalities and examines whether older LGBT people are provided with the health and care support needed for a fulfilling older age, in comparison with older heterosexual people.

Where others have combined these fuzzy concepts in previous research, the results have pointed towards disparities in the health of older LGBT people and their heterosexual counterparts. These have revealed lower levels of mental health among LGBT people (Elliott *et al*., 2015), lower levels of physical health (Elliott *et al*., 2015; Saunders *et al*., 2017; Sedlak *et al*., 2017), as well as substantial differences in health outcomes across the LGBT spectrum (Bourne *et al*., 2016). Gay men aged over 40 are less likely than younger gay men to take care of their sexual health (Williamson *et al*., 2009), less likely to have been tested for HIV/AIDS (Williamson and Hart, 2007; Knussen *et al*., 2014) and less willing to take a test (Munro *et al*., 2007). Others have examined attitudes of health and care professionals, finding substantial gaps in knowledge (Page *et al*., 2016; Willis *et al*., 2017).

Many of the studies cited above draw on Minority Stress Theory as the theoretical basis for exploring sexuality-based health inequalities. Minority Stress Theory posits that LGBT people are at an elevated risk for poorer health because of their greater exposure to social stress related to prejudice and stigma (Frost *et al*., 2015), and also provides an overall rationale for conducting this review. In this scoping review, our aim is to understand how older LGBT people experience health and care trajectories in the UK, and to what extent this experience can and should be interpreted as a health inequality compared to heterosexual people and, consequently, a health inequity. We also explore how these patterns differ across the spectrum of the LGBT acronym and across different health and care outcomes.

## Methods

The methods for this review followed the protocol published in advance of this study (Kneale *et al*., 2018). Searches were conducted on PubMed, Scopus and PsychInfo, and supplementary searches were also conducted on Google Scholar and specialist journals focused on LGBT studies. The search string was designed to be sensitive as opposed to specific, and we did not include terms reflecting health or care within the search strings. This was to ensure that general or multi-purpose studies that explored health alongside other domains of older LGBT people's lives were considered. All relevant titles and abstracts were exported into EPPI-Reviewer 4 (specialist systematic review software; Thomas *et al*., 2010) and were screened independently by reviewers (JH and DK) after an initial pilot phase to ensure consistency in screening decisions. Studies fulfilling the inclusion criteria were selected for full-text assessment, after which a new independent assessment was conducted; any disagreements were resolved through moderation between the reviewers.

Studies that were not focused on lesbian, gay, bisexual, transgender, queer or intersex older people were excluded; although no stipulation was made on the way in which LGBT status was ascertained. Studies were excluded if they were based on data collected outside the UK, were not published in English or did not focus on ‘older’ people. Being ‘older’ was notionally defined as 50+, although where studies included a mixture of ages, these were included only where the majority of participants (>75%) were aged 50+, or where data on the experiences of people aged 50+ could be disaggregated. Only studies with a focus on health or care status or needs were included, although health and care were both regarded as broad concepts involving physical and mental health and formal and informal forms of care. Studies were excluded if they did not include empirical findings about older LGBT people, and consequently commentary pieces and editorials were excluded. Case studies based on the experiences of a single subject were also excluded. Similarly, studies that did not present findings from primary research or secondary data analysis were excluded; this exclusion criteria applied to reviews and systematic reviews, although these sources were searched for relevant literature. Unlike most systematic reviews, no exclusion criteria were imposed on publication date in order to monitor change in findings over time.

Data were extracted from all included studies on study background, study design, setting, sample, data collection methods and findings. We did not undertake formal quality assessment of the studies due to the breadth of studies included (observational and experimental studies; quantitative and qualitative studies). Our methods of synthesising the data were aligned with a narrative, configurative approach (Gough *et al*., 2012). We followed five stages: (a) initial coding of the text by producing preliminary textual descriptions of studies and their findings in a tabular format; (b) further inductive coding of the textual summaries and identifying key preliminary themes and their recurrence across studies; (c) developing a framework for arranging groupings and clusters of studies according to the themes and exploration of these within and between the studies; (d) further generation of analytical themes through attempting to develop a common rubric to describe these findings; and (e) consideration of the completeness and applicability of evidence, the robustness of the analysis methods and the quality of evidence in terms of its relevance to the research question (Snilstveit *et al*., 2012). While EPPI-Reviewer was used to manage the data, NVivo (QSR International, 2017) was used to thematise and organise the data further.

## Results

After automatic duplicate checking, a total of 4,574 abstracts were screened on the basis of title and abstract, with 360 selected for full-text screening. From these, 42 studies (supported by 49 papers) were identified as meeting the inclusion criteria, and were included for further synthesis. In most cases, studies with multiple papers published from the same study were treated as belonging to the same study; exceptions occurred when studies presented data from different facets of a larger study or when the themes or data presented across papers differed substantially from one another so as to constitute a separate study. The majority of the included studies were based on qualitative methods (23 of 42); studies employing quantitative methods as the basis of the entire study (eight of 42) or part of the study in a mixed methods framework (11 of 42) were in the minority. This had implications for the generalisability of the evidence produced, with several of the studies being confined in scope to particular geographic regions or sub-regions. Many of the studies aimed to form a representative sample, although the difficulty in identifying a representative sampling frame that included sufficient numbers of LGBT people often meant that researchers resorted to sampling narrow groups of people (*e.g.* specific clinic attendees) or, more often, used snowball sampling techniques; consequently, few of the studies presented (quantitative) evidence from a representative sample of older LGBT people, or had made efforts to weight their results to account for sampling imbalances (an exception Guasp (2011), although the representativeness of the LGB sample was unclear). Most of the studies aimed to represent the ageing experience across men and women (21 of 42), although there were a greater number of studies concentrating exclusively on the ageing process among older non-heterosexual men (13 of 42) than women (eight of 42). A number of more recent studies had started to redress this balance (*e.g.* Parslow and Hegarty, 2013; Traies, 2015, 2016; Wilkens, 2016; Ingham *et al*., 2017).

In considering evidence of health inequalities and the role that being LGBT might play in shaping these inequalities, the included studies mainly drew upon narrative connecting analysis offering processual explanation to elucidate connections between health outcomes and being an older LGBT person with authors decomposing and recomposing whole events into sequentially connected social actions (Maxwell, 2004). The publication date of our included studies displays an unusual profile; no date criteria were imposed on the searches and our earliest paper was published in 1988 and focused on HIV/AIDS among older people. After this, just two papers were published in the 1990s that were UK-based empirical studies which directly collected data from older people and were focused on health and care inequalities among older LGBT people.

**Figure 1.**
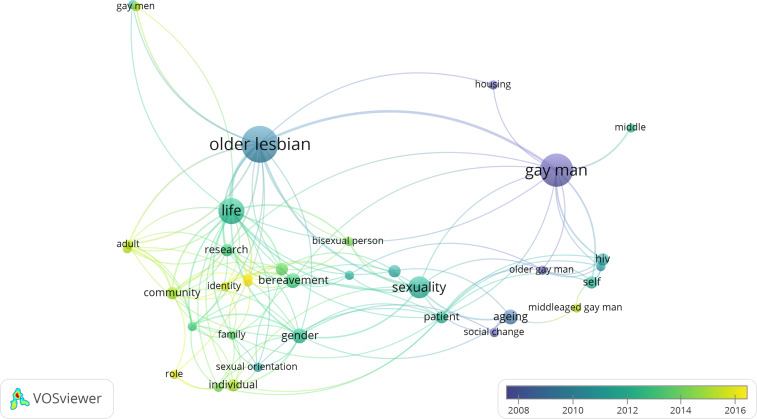
Summary of research clusters on older LGBT health and care inequalities based on title and abstract contents.

Further characteristics of all studies are described in an evidence table included in the online supplementary material which describes each study's aim, design, LGBT group included, age, sampling details, the types of inequality assessed, the type of causal account generated and a summary of findings. Furthermore, in order to help to visualise the focus of the included studies and how this has changed over time, we used bibliometric analyses to explore the co-occurrence of terms included in article titles and abstracts (van Eck and Waltman, 2009). Figure 1 demonstrates that earlier literature focused on gay men and HIV/AIDS, before moving to include explorations of older lesbian lives; more recent literature has focused on identity and community and the relationship with health, as well as the way in which age-related transitions such as bereavement can interrupt these relationships.

### Physical health inequalities and inequalities in accessing health care

A small number of studies suggested that a minority of older LGBT people were drug users (including methamphetamines, heroin and cocaine), were heavy drinkers or smoked (Bonell *et al*., 2010; Guasp, 2011; Patel *et al*., 2016). Guasp's (2011) comprehensive study on older LGBT people was one of the few included studies to compare a representative sample of older LGB people in the UK with non-LGB people, finding that older LGB people were over four times as likely to have taken drugs in the previous year (9% *versus* 2%); similarly levels of frequent alcohol consumption (five or more days a week) was 10 percentage points higher for men and 4 for women. No overall sexuality-based differences were uncovered in smoking rates, despite evidence (not included here) suggesting that LGB women of all ages are high risk of smoking-related cancers (Saunders *et al*., 2017).

Despite an overall picture presented above of older LGBT people being more likely to adopt harmful health behaviours, there were some positive health behaviours in which LGBT people were more likely to engage. For example, the proportion of older LGBT people who exercised regularly was 7 percentage points higher than heterosexual people (Guasp, 2011).

There was evidence from a number of studies that older people experienced difficulties in accessing health care that appropriately dealt with LGBT people's sexuality; this was found to be problematic among older men and women across studies (Langley, 2001; Clover, 2006; Guasp, 2011; Wilkens, 2015; Traies, 2016). In a study of men and women, Guasp (2011) found that 18 per cent of older LGBT people would feel uncomfortable disclosing their sexual orientation to their general practitione (GP) (although this was substantially lower than other care providers, *see* below), while an earlier study found that less than a third of men who had sex with men had disclosed their sexuality to their GP (Keogh *et al*., 2004). Older people maintained beliefs that health-care settings remained institutionally prejudiced, and that this perception shaped health-care usage, and sometimes directly influenced health outcomes (Guasp, 2011). For example, in Traies’ (2016) study of older women, 60 per cent reported that they had never discussed their sexuality and sexual matters with a health-care professional, although this may have reflected personal preference as well as health-care provider attitudes. Similarly, several accounts in Clover (2006: 46) reflected hostile experiences that older gay men faced in their interactions with health-care providers, with one man describing a visit to his GP after a bereavement of a partner: ‘He simply told me that if I don't feel life's worth living that's up to me what I do, which makes you feel you're worth about half a farthing, quite honestly’. Such negative interactions with health-care providers shaped the way in which older people accessed health services. However, older LGBT people may also be less likely than younger people to be able to access medical care or treatment from other sources. Bouman *et al*. (2016) found that older transgender clinic attendees were less likely than younger attendees to have accessed hormone therapy before attending clinic (*e.g.* through the internet), despite access to hormone therapy being found to have a protective effect on mental health, particularly in terms of socialisation and interpersonal problems. By extension, older transgender people who have poorer relationships with health-care providers may be expected to be particularly susceptible to these mental health issues, given that they are less likely to have access to hormone therapy from other routes.

A small number of studies focused on the experience of ageing with HIV/AIDS (Hargreaves *et al*., 1988; Elford *et al*., 2008; Owen and Catalan, 2012; Lawrence and Cross, 2013; Patel *et al*., 2016). Two of these studies emphasised that gay and bisexual men remain at risk of contracting HIV/AIDS even at older age, confounding earlier clinical wisdom (Hargreaves *et al*., 1988), with Elford *et al*. (2008) finding that a third of gay men aged 50+ living with HIV/AIDS in their study set in London had been diagnosed with the virus in their fifties. While older men were found to be more likely than younger men to be receiving antiretroviral therapy (Elford *et al*., 2008), the long-term impact of ageing while in receipt of HIV/AIDS treatment was unknown, and older men expressed trepidation at being the first cohort to be ageing while in receipt of antiretroviral therapy (Lawrence and Cross, 2013).

### Inequalities in access to social care and end-of-life care

Inequalities in access to, or perceptions of, social care was a recurring theme in 20 of the included studies. Almost all of these studies included accounts of, or perceptions of, homophobia, heteronormativity, invisibility, and a denial of older people's sexuality and identity in social care settings. Mainstream care settings were viewed as heteronormative spaces, with one participant reporting that ‘we see it as being heterosexualised, being put into a care home’ (Westwood, 2016: e157). Care environments were viewed as heteronormative and unliberated settings that required older LGBT people to assume new roles and identities (Langley, 2001). In contrast, care settings were viewed as welcoming and accepting of heterosexual relationships and sexuality (Willis *et al*., 2016). Perceptions of heteronormativity in social care were compounded further in a form of ‘double stigma’ where older people were living with other conditions such as dementia (McParland and Camic, 2018).

Anticipated and experienced heteronormativity in formal care settings was viewed as a threat to older LGBT people's identity. This manifested in different ways, including care staff's refusal to acknowledge or miscategorising of same-sex relationships (Price, 2010; McParland and Camic, 2018), to concealment of same-sex relationships (Langley, 2001; Price, 2012), to perceptions that care settings would not allow older LGBT people to express their sexuality or acknowledge relationships through, for example, displaying photos of partners (Price, 2012; Westwood, 2016). Older LGBT people expressed concern at the possibility that entering a formal care setting could lead to a ‘reversal’ of one's identity (Price, 2012; Willis *et al*., 2016; King and Stoneman, 2017). A participant included in Price (2012: 526) exemplifies this position: ‘should I need residential or nursing home care, I am not going back in the closet. I spent my life fighting to get out of the closet. I'm not going back into the closet’. Anxiety about identity concealment in formal care settings stretched across sexual and gender identity (King and Stoneman, 2017). While many older people included in studies expressed resistance to the idea that their identity would be compromised, a sizeable number may choose to conceal their identity, with almost half of older LGB people (55+) reporting that they would feel uncomfortable disclosing their sexual identity to care home staff (Guasp, 2011). Loss of identity was also expressed among older LGBT people who provided informal care to family members, where providing informal care was viewed as both a female and heterosexual endeavour (Parslow and Hegarty, 2013). Inability to express sexual identities contributed to older LGBT people's feelings of invisibility in care settings (McParland and Camic, 2018). In some cases this form of invisibility appeared to transcend individual experiences in care settings to become entrenched within policy and responses at a local authority (municipal government) level (Ward *et al*., 2008). Several studies reported that older people anticipated or experienced active homophobia in care settings (Smith, 1992; Heaphy *et al*., 2004; Almack *et al*., 2010; Phillips and Knocker, 2010; Guasp, 2011; Lawrence and Cross, 2013; Westwood, 2016, 2017*b*, 2017*c*; Willis *et al*., 2016; King and Stoneman, 2017; McParland and Camic, 2018). Studies suggested that older LGBT people could avoid coming into contact with homophobic individuals in the general community in a way which they could not in formal care settings (Westwood, 2017*c*). These studies emphasised that the loss of autonomy often associated with ageing has a disproportionately negative impact on sexual minorities.

Some studies suggested that the social networks of older lesbian and gay people were structured differently compared to those of non-LGBT people which may increase the risk of requiring formal, as opposed to informal, care (Heaphy and Yip, 2003; Almack *et al*., 2010; King and Stoneman, 2017; Westwood, 2017*b*). For example, Heaphy and Yip (2003) describe how some older people who may have been estranged from their families, and who may have failed to develop strong friendship networks, could regard their future prospects with regards to care as bleak. An absence of children was viewed as placing LGBT people in a precarious position in terms of future care, with some older people reporting that ‘one doesn't have a younger generation of family to fight your corner should you be unable to do it for yourself’ (Guasp, 2011: 9). Nevertheless, studies also included accounts from older LGBT people who described increasing possibilities and complex configurations of biological and social kin that formed networks that could potentially maintain their independence for longer (Heaphy and Yip, 2003; Almack *et al*., 2010).

A number of studies emphasised that the hopes and fears of older LGBT people relating to access to care in later life mirrored those of non-LGBT people (Langley, 2001; Almack *et al*., 2010; Parslow and Hegarty, 2013; McParland and Camic, 2018). These included fears around the loss of independence and autonomy, and around dementia, entering formal care settings, and a fear of dying alone. However, these fears were often exacerbated in the case of older LGBT people through experiences of homophobia, transphobia, heteronormativity, invisibility, and denial of identity described above. Many studies described strong communities and networks of older LGBT people as being important in helping older people to maintain their independence (Wilkens, 2015; Simpson, 2016; King and Stoneman, 2017), and participants included in some studies expressed a wish for some of these networks to become more structured. For example, Owen and Catalan explored HIV-positive gay men and recount one participant's aspiration of:

getting into a supportive community. Obviously a bit of the old hippie in me and a kind of romantic idea maybe, but a big rambling house with a nice big kitchen and a place where we can eat together but have our own areas too. (Owen and Catalan, 2012: 68–69)

In some studies this extended to a desire for separate formal care settings for LGBT people away from non-LGBT people (Lawrence and Cross, 2013; Traies, 2016; Westwood, 2016; King and Stoneman, 2017). Lawrence and Cross (2013) found this to be a common aspiration among older HIV-positive gay men; meanwhile Traies (2016) found that the overwhelming majority of older lesbian women in her research (composed of a large sample of 350+) were positive about lesbian-only care homes, in contrast to the highly negative ratings given for mixed homes. Nonetheless, this was not a universal finding across studies, with other participants and studies describing older LGBT people resistant to the notion of being ‘ghettoised’ in specialist LGBT care settings, and instead wishing to remain part of a broader community (Price, 2012; Westwood, 2016). Studies considering the provision of home care also suggested that gender and sexual identity of visiting carers was important for many older LGBT people, although this differed across the spectrum, with older gay men likely to place more importance on the sexuality of their carer than their gender, while older lesbian women placed greater importance on the gender of their carer than sexuality (King and Stoneman, 2017).

The treatment of older LGBT settings described in the studies is broadly suggestive that social care settings are a focal point for health and care inequalities. The consequences of such treatment may be that older LGBT people are more likely to ‘dread’ accessing care services (Langley, 2001; Almack *et al*., 2010; Phillips and Knocker, 2010), and may consequently be less likely to plan care transitions (Heaphy *et al*., 2004), increasing the risk that any future transitions to formal care settings are both stressful and disorganised. For example, despite concerns about possible experiences of homophobia and transphobia in care settings, only 72 per cent of older LGBT people in a recent study had taken any steps in planning their future care (King and Stoneman, 2017).

A smaller set of studies explored inequalities in the provision of end-of-life care and bereavement support, uncovering similar themes as above. Here the invisibility and denial of identity discussed above were expressed as disenfranchisement of grief, where the catastrophic loss of a partner was trivialised by care providers, as well as wider social networks and families (Fenge and Fannin, 2009; Almack *et al*., 2010; Piatczanyn *et al*., 2016; Ingham *et al*., 2017). End-of-life care and bereavement were particularly difficult experiences where older LGBT people had not disclosed the nature of a relationship before an illness, and where bereavement could also necessitate coming out for the surviving partner (Fenge and Fannin, 2009; Almack *et al*., 2010). As was the case above in terms of access to social care, the evidence suggested that the provision of specialist care was lacking (Fenge and Fannin, 2009), and that mainstream end-of-life care and bereavement support providers were ill-equipped to deal with the needs of older LGBT people (Fenge and Fannin, 2009; Almack *et al*., 2010).

### Experiencing loneliness, social isolation and mental health problems

Loneliness and social isolation was not a universal experience for older LGBT people. Older people who were able to form relationships and social networks, particularly with other older LGBT people, were cushioned from feeling isolated and lonely, and could draw on emotional, social and economic resources from others to navigate crises or age-related transitions (Langley, 2001; Heaphy and Yip, 2003; Heaphy *et al*., 2004; Price, 2012; Cronin and King, 2014; Traies, 2015; Wilkens, 2015, 2016). While these networks appeared to have formed organically in some accounts (Heaphy *et al*., 2004), in others they were more structured in the form of LGBT-specific groups, which were viewed as valuable in many studies in creating social connections between older LGBT people (Phillips and Knocker, 2010; Price, 2012; Traies, 2015; Wilkens, 2015, 2016). This was articulated by a participant in Price as a respectful acknowledgement of difference and providing an opportunity to explore and connect over this difference:

For example, I've just been out on my first trip with the Gay Birdwatching Club, a national group of birders who go out at weekends together. Anyone might say well you can birdwatch in any group of like-minded souls – but there's something about being in a majority, sharing a culture, not having to explain, having the same reference points, etc. etc., which straight people never even think about because for them it's the norm. We should not have to justify wanting this contact with other gay people other than to say we enjoy it! (Price, 2012: 525)

Conversely, where these resources did not exist or were not accessible, inadequate resources for older LGBT people to meet and socialise were linked with increased risks of social isolation and loneliness in several studies (Langley, 2001; Clover, 2006; Fenge and Fannin, 2009; Phillips and Knocker, 2010; Price, 2012; Cronin and King, 2014; Wilkens, 2015; King and Stoneman, 2017). Barriers to developing social (and sexual) relationships with other LGBT people included family obligations as well as socio-economic resources (Heaphy, 2009).

Creating new social, romantic and sexual networks as an older lesbian or gay person was challenging. For several older gay men, the HIV/AIDS epidemic had a devastating impact on their friendship networks, leaving substantial gaps and a feeling of premature ageing (Phillips and Knocker, 2010; Owen and Catalan, 2012). LGBT-specific groups and spaces were viewed as difficult to access for older people who had recently lost a partner and who found it difficult to reconnect and ‘start over’ with non-heterosexual networks (Traies, 2015). In the case of commercial venues, particularly for gay men, they were viewed as youth-orientated or actively ageist (Heaphy and Yip, 2003; Owen and Catalan, 2012; Simpson, 2013; Cronin and King, 2014; Piatczanyn *et al*., 2016), compounding difficulties in (re)connecting.

Older LGBT people described challenges in presenting as an LGBT person in developing (new) heterosexual social networks (Heaphy and Yip, 2003), reporting difficulties in finding common ground over subjects such as children and grandchildren (Cronin and King, 2014; Wilkens, 2016). In other cases, older people described manifestations of internalised homophobia and minority stress in creating or maintaining heterosexual networks, being unable to present as their authentic selves in heterosexual circles and leading a double life (Heaphy and Yip, 2003; Simpson, 2013; Cronin and King, 2014). For some older people, limitations in social networks were also mirrored by unsupportive (biological) family relationships, compounding feelings of isolation and creating concern about how future age-related transitions would be managed (Westwood, 2017*b*).

Limited social networks, experiences of social isolation and feelings of intense loneliness manifested as physical pain for some older LGBT people: ‘I feel completely isolated a lot of the time … I desperately try and at least talk to somebody, talk to a lesbian every day … I've got loads of friends on the periphery, um, but yeah I often feel, oh physical pain, I feel so alone’ (Wilkens, 2015: 95). This type of loneliness may be felt by a sizeable minority of older LGBT people, with one in 12 older LGBT people in one study reporting that they had no one they could turn to for emotional support (King and Stoneman, 2017). Other studies also pointed to elevated levels of mental health issues among older LGBT people. Bouman *et al*. (2016) examined the mental health of older transgender women, finding that 16.9 per cent of attendees to gender reassignment clinic reported non-suicidal self-injuries; around three times higher than average among the general population. The risk of suicidal ideation was found to rise with age among older bisexual women, with the authors describing how older bisexual women faced ‘double discrimination’ (Colledge *et al*., 2015), and could in fact have faced three types of discrimination on account of their gender, age and sexuality. Suicide was also found to be prevalent in the narrative histories of many older LGBT people in rural areas, where internalised homophobia, as well as fear of or actual experiences of being involuntarily ‘outed’ to hostile communities, were attributed as triggering suicide (Jones *et al*., 2013).

Despite most studies suggesting a link between older LGBT people and a higher risk of mental health issues, one study found that 16 per cent of older LGBT people completing the General Health Questionnaire (GHQ-28) had scores indicating psychological distress (Warner *et al*., 2003); this is a similar level to that found in a sample of the general population aged 53 years (Hatch *et al*., 2009). However, the snowball recruitment methods employed by Warner *et al*. (2003) mean that the results of this particular study should be interpreted with caution.

### Inequalities – experiencing violence

In addition to the experiences of heteronormativity and homophobia experienced within health and social care settings, a number of the studies included additional details of homophobia, aggression and violence that older people faced in their day-to-day lives. This ranged from aggression that followed expressions of affection between same-sex partners (Simpson, 2013), to less-specific incidents that prompted older LGBT people to conceal their identities (Heaphy and Yip, 2003; Heaphy *et al*., 2004; Lawrence and Cross, 2013; King and Stoneman, 2017). Anxiety around aggression and micro-aggression was a particular concern of older transgender people, as expressed by one transgender woman in King and Stoneman (2017: 93): ‘I think in housing terms you are having to think about who are your neighbours and how are your neighbours going to act, and are your neighbours going to cause you problems?’

One study described the ‘psychiatric treatment’ that older gay men and transgender women had received earlier in their lifecourse that involved sustained and severe degrees of physical and mental violence (Dickinson *et al*., 2012). Despite several of the participants having accepted their current situation, the traumatic treatment they experienced continued to shape their relationships, mental health and patterns of access to health care (Dickinson *et al*., 2012); an identical experience was also relayed in Hubbard and Rossington:

I was first sent to see a psychiatrist in 1925 when I was 17 years old. I remained under treatment for 25 years receiving aversion therapy and drug treatment. This includes two periods as an inpatient. In 1950 I was discharged and told I was incurable. It is only in the last 5 years that I have felt good about myself and my sexuality. (Hubbard and Rossington, 1995: 8)

## Summary and discussion

### Generalisability of evidence

This review finds evidence suggesting that health inequalities, or more specifically health inequities, exist in the health and care status of older LGBT people and, in particular, in the opportunities available for older LGBT people to plan and access health and social care that is stigma-free and accommodating of different health and care needs. Much of this evidence is drawn from qualitative studies that have uncovered similar themes, and where the results can be considered analytically generalisable (Pratt, 2008; Leung, 2015) in that they can be generalised to the Minority Stress Theory. However, while as a body of evidence these studies may hold analytical generalisability, the body of evidence had low levels of ‘proximal generalisability’ to several groups of older LGBT people. These included older LGBT people living in Wales, Scotland and Northern Ireland and many parts of England (*e.g.* North-East England), older people from poorer backgrounds, older LGBT people from Black and Minority Ethnic backgrounds, but perhaps most prominently, older transgender people who were absent in the majority of studies.

In terms of quantitative studies, none of the studies offered evidence drawn from the analyses of data collected through random probabilistic sampling that could be generalised beyond the immediate confines of the sample, or could only be generalised beyond narrow groups or experiences (*e.g.* clinic attendees). However, invisibility and exclusion of older LGBT people may have meant that probabilistic sampling may have been unsuitable in trying to generate samples with sufficient numbers of older people to support analyses. A study that did include a heterosexual comparison group, and potentially is closest to providing evidence that is generalisable to a broader population due to the large sample size of LGB people included in analyses (1,036 LGB men and women) found that LGB people were more likely to be frequent drinkers of alcohol and to report poorer mental health than heterosexual people, but no overall differences were found in smoking rates, and one health advantage was uncovered with LGB older people reporting exercising more frequently than heterosexual people (Guasp, 2011). This particular study was limited in other ways, however (*e.g.* a failure to account for potential confounders).

### Summary of evidence and analytical generalisability

Experience of homophobic discrimination was a theme across most studies, both earlier in the lifecourse and on new grounds, particularly in social care settings, while expressions of internalised homophobia were also reported in a number of studies, for example through denial of identity (Cronin and King, 2014). In line with the Minority Stress Theory, these experiences raised the risk of mental health problems, and we outline in Figure 2 the possible routes towards an increase in care needs that these experiences triggered. For example, a study by King and Stoneman (2017) clearly outlines how discrimination directly impacts on the social connections of LGBT people which consequently has an impact on the breadth of their care choices. As single studies that are predominantly reliant on convenience sampling, the generalisability of such evidence could be overlooked because of the methods used within these studies. However, through examining the body of literature as a whole, we consider the findings presented here to be analytically generalisable, and they indicate that:

1. Social care environments in particular appear as a nexus for inequities in the health and care of older people. Formal care environments actually or were perceived as severely compromising the identity, relationships and life that older LGBT people had developed. Some LGBT people faced spending later life in homophobic social care environments, providing the very antithesis of ‘care’ (theme supported by 20 studies).

**Figure 2.**
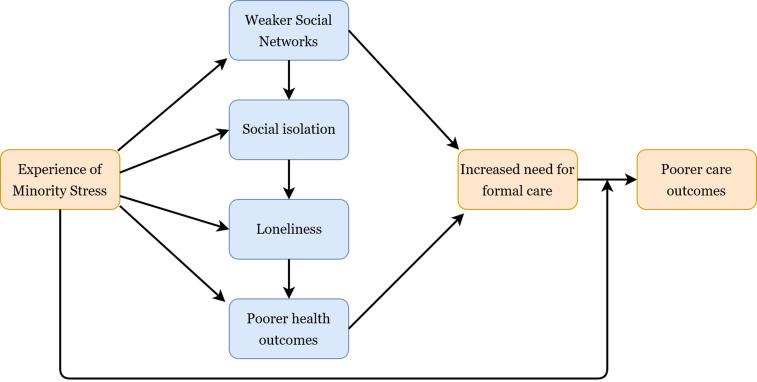
Logic model summarising the review findings: minority stress and health and care inequalities among older LGBT people.
2. Experience of minority stress places pressure on older LGBT people's social connections and networks (theme supported by four studies).
3. Weaker social networks directly raise the risk of requiring formal social care because of reduced options for informal care (theme supported by five studies).
4. Weaker social networks raise the risk of social isolation and loneliness (theme supported by ten studies), which may also indirectly serve to raise the risk of requiring formal care.
5. A number of studies document experiences of homophobia in interactions with health and care providers earlier in the lifecourse among older LGBT people. This may shape current and future interactions and expectations of service with health and care providers (theme supported by six studies).

Overall, the evidence suggests that (unchecked) minority stress raises the risk of older LGBT people needing social care, and within formal care environments older LGBT people face high levels of heteronormativity and homophobia that increases the risk of poorer care outcomes, as summarised in the logic model in Figure 2. Similar pathways are also observed in clinical health-care settings (Fish and Williamson, 2018). Recognition of these risks is not commensurate with adopting a ‘victim trope’ (Farrier, 2015) and older LGBT people are not pre-destined to follow these deleterious trajectories. Instead, much of the literature also focuses on how older LGBT people can navigate age-related transitions and avoid some of the age- and sexuality-related challenges. For example, a number of the studies examined the way in which LGBT-focused social groups are particularly impactful for LGBT people in offsetting feelings of isolation and loneliness, maintaining independence and meeting new partners (Phillips and Knocker, 2010; Wilkens, 2015, 2016). Nevertheless, in order to create enabling environments that promote active ageing for LGBT people, evidence is needed that demonstrates the inequalities that older LGBT people face. This scoping review arguably presents strong evidence of health and care inequities, although, as discussed below, the evidence is less conclusive on the magnitude of these ‘inequalities’.

### Additional strengths and limitations

This review presents a review across the health inequalities among older LGBT people. In identifying the literature, a broad search, which increased the screening burden, was implemented across the main databases in order to identify a comprehensive set of studies that examined the health of older LGBT people in the UK. Studies using a broad range of methodologies and theoretical standpoints, and which explored trends among a diverse group of people, were included. This provides a comprehensive picture of LGBT health and care experience in the UK. However, some caveats do apply.

The review had a narrow geographic scope, being confined to UK studies alone. While this allows us to assume understanding of the legislative landscape, as well as the way in which older people access health and social care, all of which shape the emergence of health inequalities, this may pose limits on the generalisability of the findings. Similarly, our imposition of an age criteria of 50+ may mean that studies which could shed light on ageing processes were missed. For example, Sedlak *et al*. (2017) explored osteoporosis knowledge and avoidance strategies among transgender people, finding that despite their elevated risk through long-term hormone use, transgender people exhibited poor knowledge of osteoporosis. In contrast, a study by Maylor *et al*. (2007) suggested that sexuality played little part in the way in which cognition decreased with age. Both studies were excluded because of the age group and location of the participants. While these examples do not change the conclusions of the review, such examples could provide further nuance to this complex body of evidence. We also imposed a restriction that studies needed to collect data directly from older people themselves, and consequently excluded studies that focused on people who work with older LGBT people. Studies that examined the ‘causes of causes’ of ill-health or elevated care needs were also excluded. For example, studies that focused on the social networks of older LGBT people, but did not present direct evidence of how these translated to increased risks of social isolation/loneliness or risks of entering formal care, were not included (*e.g.* Kneale *et al*., 2014; Green, 2016; Kneale, 2016).

Methodologically, the review included a breadth of study designs, which shaped our decision not to formally quality assess the studies. This meant that studies that may have a high risk of bias could have contributed to the findings and shaped the conclusions in a way in which we are unable to take account of. One of the main limitations of the review is our decision to pool evidence for LGBT people together. This is despite some of the theories reflecting health and care needs, or indeed minority stress, pulling in separate directions across the LGBT acronym. For example, the theory of accelerated ageing may operate in different directions for gay men as it does for gay/lesbian women, which may have an impact on health in later life (Laner, 1979; Wight *et al*., 2015). The breadth of the review means that the results are theory generating, exploring how minority stress shapes later-life health in the UK, but may overlook important nuances in the evidence. Nevertheless, the nature of the systematic scoping review is to give a grounding for future review and research activity, and in this respect the review provides a useful basis for future work in this area. Areas where future researchers may beneficially focus their efforts include the health and care inequalities faced by older transgender men and women, who were almost entirely absent in the literature. Studies such as those conducted by Bouman *et al*. (2016) and King and Stoneman (2017) help to address this gap partially through having an exclusive focus on transgender people or through disaggregating information on transgender people, although there remain substantial gaps. Earlier studies highlighted the invisibility of older lesbian, gay and bisexual women in research (Westwood, 2017*a*), with much earlier studies highlighting that lesbianism among older women used to be regarded as a ‘proclivity of a small, adventurous, minority’ (Kehoe, 1986: 140). This review finds a number of studies that are focused exclusively on older LGB women, although this pool of literature needs to grow substantially if it is to compensate for the years of neglect in this area. Substantive gaps were also found in terms of evidence on physical and mental health inequalities faced by older LGBT people, and how other intersectionalities including class, ethnicity and areas of residence interact with sexuality-based inequalities.

Finally, had we implemented quality assessment criteria, we may have concluded that the evidence base poorly supports the study of health and care inequalities for four main reasons. Firstly, many of the studies draw upon narrative-connecting analysis to make a link between the sexuality and health inequalities. Few studies were able make explicit comparisons between LGBT people and heterosexual people, and some of the sexuality-based inequalities were inferred in the studies. Qualitative studies (without a comparison group) can be used to create powerful causal narratives on the role of sexuality in defining sub-optimal health and care trajectories, as was the case here. However, in the case of exploring health inequalities, without evidence that makes direct comparisons with heterosexual people's health, the body of evidence as it currently stands provides stronger evidence of health inequities (*i.e.* injustices in treatment) than it does inequalities (allowing for understanding the magnitude of disparities in health status) *per se*. In other words, many of the included studies allowed us to understand and theorise some of the mechanisms by which disparities in health and care might arise, but not directly to observe their impact on differences in health or care status. Secondly, the body of evidence that employed quantitative methods mainly did so with samples that were not generated through representative sampling frameworks, and did not weight the results to attempt to ensure representativeness. Where a comparative sample was drawn, and weights used, there was no attempt to control for potential confounders, again compromising the validity of the results. Thirdly, few reports of interventions or trials were discovered and included, despite their potential eligibility. Again, while such interventions may not include a comparison group of non-LGBT people, as the programme theory may have an LGBT-specific design, a more robust design measuring change between pre- and post-intervention may provide more compelling evidence for decision-makers on the malleability of health inequities to interventions. Finally, we described in the introduction the way in which the legislative landscape has become more permissive in opening up freedoms for LGBT people, although the social landscape has lagged behind. However, none of the studies exploring health inequalities have directly addressed the impact of policy changes on health and care inequalities through, for example, longitudinal designs or repeated cross-sectional designs. This appears to be an omission in the evidence base, and longitudinal studies appear to be an area where further investment is needed.

## Conclusions

The main contribution of this review is to provide a comprehensive synthesis of the evidence and we conclude that older LGBT people in the UK do experience inequities in their health and care status. Our synthesis is broadly consistent in showing that formal care environments, and the provision of informal and formal care more generally, emerge as a nexus for the emergence of health and care inequalities. The literature suggests that for some older LGBT people, weaker social networks, higher risks of isolation and loneliness, and poorer health behaviours accelerate a need for formal care; however, they may be the very people to face unequal treatment in these environments (Figure 2). Identification of this model based on the evidence is a contribution of this review and was only made possible through synthesising evidence on physical health, mental health and social care simultaneously, areas that are often regarded as distinct in policy and practice. Furthermore, the evidence suggests that although many older LGBT people have a fear of care settings, only a minority plan key elements of later life, such as housing choices, that could help to offset the need for entering formal care institutions. However, we know little about when and why the health status of LGBT people changes and triggers a need for entering formal care environments. A key area of omission in the focus of research on older LGBT people appears to be understanding the extent to which health inequalities manifest in terms of physical health and general health status. For example, there exists an evidence gap with respect to even the most rudimentary measures of health status such as self-rated health or presence of long-term illness.

This review also adds to the debate about how services for older LGBT people should be planned and delivered in the UK. Earlier researchers had speculated that older LGBT people would provide a role model to other older people on the navigation of age-related transitions and age-related stigma. Lee draws on the work of Bergen who described that among older gay men:

The older homosexual mastered … a crisis of being stigmatized: He learned to manage an identity that was in disfavour almost everywhere. A similar crisis of being stigmatized characterizes passage into the status of older age for all men and women … Studying the coping mechanisms used by homosexuals to deal with their stigma may shed light on how the elderly can learn to manage the stigma of old age. (Lee, 1987: 54)

This review confirms that, left unchecked, the stigma that older LGBT people may have faced in their earlier years is encountered again in predominantly heteronormative, and often homophobic, care environments. Experience of, or anticipation of this stress, leads to poorer health and social care outcomes. The resilience that authors such as Bergen may have been describing can be drawn upon most effectively within enabling environments, and some of the studies included in this review point to the type of support that appears to help older LGBT people to offset these inequities, including predominantly LGBT-focused social groups (Phillips and Knocker, 2010; Wilkens, 2015, 2016; Traies, 2015). However, the evidence is at best ambiguous on whether care services for LGBT people should be specialist or integrated into mainstream services. For example, LGBT-specific care is an attractive option to many older LGBT people, while others would prefer mixed but supportive environments. Meanwhile, for some transgender older people, LGBT-focused environments themselves could be perceived as being equally hostile as mixed environments due to transphobia from within the LGBT community (King and Stoneman, 2017). The overwhelming message from this body of literature is the need for diversity in care options in later life, and a common standard around the provision of knowledgeable and respectful care for older LGBT people.

While it is important to recognise the innate resilience that many older LGBT people exhibit in navigating older-age transitions, it is essential to develop a greater understanding of the health and care challenges that older LGBT people face in order to create the types of environments that support older LGBT people. While Minority Stress Theory is clearly an underlying explanatory framework of how inequities may arise, evidence demonstrating the way in which these experiences shape ageing trajectories and health and care inequalities in the UK remains somewhat elusive. The evidence in this review suggests that social care is a nexus for health inequities for older LGBT people; while these environments need to change in order to accommodate an increasingly diverse older population, the question we should all consider next is how to support older LGBT people to maintain their independence for longer, reducing the need for formal care.

## Supplementary material

For supplementary material accompanying this paper visit https://doi.org/10.1017/S0144686X19001326. click here to view supplementary material
