# Supplementary material for: Inequalities in older LGBT people’s health and care needs in the United Kingdom: a systematic scoping review
Source: Ageing Soc. Author manuscript; Available in PMC 2024 Dec 7. (PMC8423450; doi:10.1017/S0144686X19001326)
Supplement: Supplementary - Exclusion critieria [file EMS85345-supplement-Supplementary___Exclusion_critieria.docx]

# Supplementary materials

**Table 1: Exclusion criteria**

| - **Exclude 1 - Duplicate** - **Exclude 2 - Not about UK**   *Does not focus on setting(s) in any part of the UK*   - **Exclude 3 - Not about LGBTQ**   *Does not include Lesbian, Gay, Bisexual or Transgender people as a focus or comparative focus*   - **Exclude 4 - Not older LGBT**   *Does not include older people aged 50+ as a focus or comparative focus*   - **Exclude 5 - Not about health or care**   *Does not include a focus on health or care needs of older LGBT people*   - **Exclude 6 - Not empirical or is a case study of individual**   *Is not empirical (e.g. a theoretical study)*   - **Exclude 7 - Exclude on Publication Type**   *Not a primary study: is a review piece, systematic review, letter or commentary*   - **Exclude 8 - Not English**   *Is not available in English*   - **Include for Full Text** |
| --- |
