# Supplementary material for: Inequalities in older LGBT people’s health and care needs in the United Kingdom: a systematic scoping review
Source: Ageing Soc. Author manuscript; Available in PMC 2024 Dec 7. (PMC8423450; doi:10.1017/S0144686X19001326)
Supplement: Supplementary - PRISMA [file EMS85345-supplement-Supplementary___PRISMA.docx]

Studies included in synthesis
(n = 42)

Papers included in synthesis
(n = 49)

Full-text articles excluded, with reasons
(n = 322 )

Duplicate or linked study (n=11)

Based outside UK (n=209)

Not LGBT (n=4)

Information not directly collected from older LGBT (n=42)

Not about health or social care (n=17)

Not empirical or case study of single individual (n=38)

Not in English (n=1)

Records excluded
(n = 4162)

Records screened on title and abstract
(n = 4526)

Full-text articles assessed for eligibility
(n = 364 )

Records after duplicates removed
(n = 4525)

Additional records identified through other sources
(n = 4)

## Identification

## Eligibility

## Included

## Screening

Records identified through database searching
(n = 5738)
