# Supplementary material for: Inequalities in older LGBT people’s health and care needs in the United Kingdom: a systematic scoping review
Source: Ageing Soc. Author manuscript; Available in PMC 2024 Dec 7. (PMC8423450; doi:10.1017/S0144686X19001326)
Supplement: Supplementary - Search syntax [file EMS85345-supplement-Supplementary___Search_syntax.docx]

# Supplementary materials – example search syntax

Search syntax for Scopus:

( ( ( TITLE-ABS-KEY ( lgbt )  OR  TITLE-ABS-KEY ( lesbian )  OR  TITLE-ABS-KEY ( gay )  OR  TITLE-ABS-KEY ( queer )  OR  TITLE-ABS-KEY ( bisexual )  OR  TITLE-ABS-KEY ( transgender )  OR  TITLE-ABS-KEY ( transsexual )  OR  TITLE-ABS-KEY ( homosexual )  OR  TITLE-ABS-KEY ( intersex ) ) ) )  AND  ( ( TITLE-ABS-KEY ( ageing )  OR  TITLE-ABS-KEY ( aging )  OR  TITLE-ABS-KEY ( older )  OR  TITLE-ABS-KEY ( elder )  OR  TITLE-ABS-KEY ( aged ) ) )  AND  ( ( ( ( TITLE-ABS-KEY ( uk )  OR  TITLE-ABS-KEY ( united  AND  kingdom )  OR  TITLE-ABS-KEY ( england )  OR  TITLE-ABS-KEY ( english )  OR  TITLE-ABS-KEY ( britain )  OR  TITLE-ABS-KEY ( british )  OR  TITLE-ABS-KEY ( wales )  OR  TITLE-ABS-KEY ( welsh )  OR  TITLE-ABS-KEY ( scotland )  OR  TITLE-ABS-KEY ( scottish )  OR  TITLE-ABS-KEY ( ireland )  OR  TITLE-ABS-KEY ( irish ) ) ) )  OR  ( ( ( AFFIL ( uk )  OR  AFFIL ( united  AND  kingdom )  OR  AFFIL ( england )  OR  AFFIL ( english )  OR  AFFIL ( britain )  OR  AFFIL ( british )  OR  AFFIL ( wales )  OR  AFFIL ( welsh )  OR  AFFIL ( scotland )  OR  AFFIL ( scottish )  OR  AFFIL ( ireland )  OR  AFFIL ( irish ) ) ) ) )
