# Supplementary material for: Inequalities in older LGBT people's health and care needs in the United Kingdom: a systematic scoping review
Source: Ageing Soc. 2019 Oct 11;41(3):493–515. doi: 10.1017/S0144686X19001326 (PMC8423450; doi:10.1017/S0144686X19001326)
Supplement: Supplementary file 1 [file S0144686X19001326sup.zip › S0144686X19001326sup002.docx]

| **Short Title** | **Aim** | **Study design** | **LGBT population** | **Sample age and further details** | **Types of inequality assessed** | **Type of causal account** | **Summary of inequality** |
| --- | --- | --- | --- | --- | --- | --- | --- |
| **Almack, Seymour and Bellamy (2010)** | Explores how sexual orientation impacts on concerns about, and experiences of, end of life care and bereavement. | Qualitative Focus group: four focus groups (and two follow-up groups with some of the same participants) lesbian and gay elders (N = 15) | Gay men; Bisexual men; Gay or Lesbian women; Bisexual women | n=8 aged between 55 and 74; n=1 aged 75–84; n=1 aged <45 (a support worker); n=5 did not give their age. Groups conducted in York and London | Access to social care Access to end of life care | Narrative connecting analysis: e.g. Links made between previous experiences and fears for the future in care settings | Age-based inequalities among LGBT people considered where levels of social isolation may be further exacerbated by ageism within LGB communities. Sexuality-based inequalities described through interpretation of homophobia and minority stress etc. |
| **Bonell, et al. (2010)** | Examine: patterns of use of methamphetamine and across specific subgroups | Quantitative Cross-sectional section survey including 694 responses from men aged 50+ | Described as gay men in the interpretation; sample frame included men who were gay, bisexual or had another non-heterosexual identity | 50+ represented as a group; examined patterns in London vs other regions. | Drug use (Methamphetamine use) | Theoretical or mechanistic evidence provided to support higher levels of drug use is higher among gay men | Compared younger and older gay men; older gay men have lower risk of methamphetamine use; although this lower level may still be higher than among peers of same age. |
| **Bouman, et al. (2016)** | Investigated the sociodemographic and clinical characteristics of older trans people and investigated the influence of cross-sex hormones (CHT) on psychopathology. | Quantitative Survey based on clinic attendees | Transgender Women (M2F; n=71) (a small number of trans males were removed for the analysis due to their small number (n=3). | Mean age was 58.9 years. Attendees of East Midlands clinic | Access to healthcare; Patterns of Mental health | Elements of counterfactual reasoning - access to hormone therapy vs not | Suggestion of age-based inequalities within LGBT community with older trans women less likely to have accessed hormone therapy; sexuality-based inequalities implicit through focus on access to hormones and comparatively high levels of self-harm reported in the sample. |
| **Clover (2006)** | Aim to explore the experiences of older gay men in relation to health | Qualitative: semi-structured interviews with ten age men | Gay men (described as gay by author) | All aged 60-70 | Access to healthcare Loneliness/social isolation | Mainly provided narrative connecting analysis and some logical reasoning - the men described that when they revealed they were gay, they immediately received poor treatment. Some evidence from discrepant experiences of good treatment and the mechanisms described | Sexuality-based inequalities in access to health care (free from discrimination) implicit. Earlier experiences may impact health care utilisation in later life. |
| **Colledge, et al. (2015)** | Seek to address an evidence gap around mental health differences between lesbian and bisexual women. | Quantitative: Self-completion survey with community-based, opportunistic sampling | Gay or Lesbian women; Bisexual women; a number identified as trans. | 219 women aged 50+ years included | Mental health | Theoretical or mechanistic evidence provided to explain why ‘older bisexual women may have experienced greater sexuality-related ‘double discrimination’ and internalized stigma than younger bisexual women, having adopted their sexual identity at a time of greater social stigmatization of bisexuality’ including drawing on Minority Stress Theory | Sexuality-based inequalities apparent through high levels of mental health issues including suicidal ideation (not all numbers disaggregated by age). Age-based inequalities to suggest that sexuality-based inequalities are particularly severe among older women. |
| **Cronin and King (2014)** | Aimed to examine social factors that mediate access and participation in lesbian and gay communities | Qualitative: Semi-structured interviews | Gay men; Bisexual men; Gay or Lesbian women; Bisexual women | All aged 50-73 years; n=22 adults (n=11 men; n=11 women) | Loneliness/social isolation | Narrative connections based on theoretical and narrative analysis of interviews | Sexuality-based inequalities were implicit; the study presented a nuanced picture around the importance of LGB networks for older people, which were found to be protective against the stresses of living in a heteronormative society. Some age-based inequalities also implicit and described with regards to ageism within commercial gay venues. |
| **Dickinson, et al. (2012)** | Aimed to examine the experiences of patients and meanings attached to ‘treatments’ of sexual deviations, which included homosexuality and transvestism, in historic psychiatric settings and the outcomes of such treatment. | Qualitative: A nationwide study based on oral history interviews. | Gay men and Transgender Women | Seven former male patients were recruited, aged from 65–97 years at interview. N=5 were treated for homosexuality; n=2 were treated for transvestism and were living as females. | Violence | Narrative connecting analysis providing a powerful summary of the climate of violence LGBT people faced (particularly males). Implications are that many older LGBT people are reluctant and fearful to seek mental health care services because of historical treatment. | Sexuality-based inequalities are clear and the study is demonstrative of the climate of violence facing older LGBT people during their younger years and how this may continue to shape patterns of health and social care access in the future. |
| **Elford, et al. (2008)** | Sought to examine age at diagnosis, sexual behaviour and some social characteristics of older people living with HIV (with particular focus on gay men) | Quantitative Survey  of clinic attendees with HIV infection in an outpatient clinic in London. | Gay and bisexual men | The sample included 99 gay and bisexual men aged 50+. (majority aged 50-59 years, with relatively few aged 60 years or more; the oldest was 72 yrs old) | HIV/AIDS | Some theoretical and mechanistic evidence presented around the added pressure of living with HIV/AIDS in older age. | Sexuality-based inequalities are implicit with the higher risk of contracting HIV/AIDS among men who have sex with men. High levels of depressive symptoms self-reported. Older HIV positive men exhibited similar risk behaviours around sero-sorting and unprotected sex to younger HIV+ve men. |
| **Fenge and Fannin (2009)** Fenge (2008) | Explores the impact of sexuality on the experience of bereavement in later life | Mixed methods – action research project | Gay men; Gay or Lesbian women (bisexual and transgender not mentioned although would be eligible) | Eligible ages broadly 55-85 years | Access to healthcare Loneliness/social isolation Access to end of life care | Processes and experiences of being gay/lesbian and the impact on bereavement and support narratively connected | Sexuality-based inequalities in the support available for the surviving partners of same-sex relationships identified. |
| **Guasp (2011)** | Compared experiences and expectations of getting older among LGBT vs non-LGBT people, focusing on personal support structures, family connections and living arrangements | Quantitative: study (note commercial provider) | Gay men; Bisexual men; Gay or Lesbian women; Bisexual women | Aged 55+ | Drug Use Smoking Exercise Mental health Access to healthcare Access to Social Care | Counterfactual reasoning – the survey includes a heterosexual group of a similar age and strength of associations evaluated (confounders not addressed) | Sexuality-based inequalities are evidence across a range of health and care areas including mental and physical health domains. Poorer relationships with health and care providers are also described. |
| **Hargreaves, Fuller and Gazzard (1988)** | Case study of two patients, aged 64 and 73, who presented with chest infections; the diagnosis of AIDS was initially overlooked on the basis of both their age and the history. | Qualitative Case study | Gay men (MSM) | Aged 64 and 73 years | Access to healthcare HIV/AIDS | Unclear although to some extent the paper offers evidence of reversibility as when treatment was applied to one of the patients, which was previously denied because of age/sexuality, their condition improved | Sexuality-based inequalities are implicit with the higher risk of contracting HIV/AIDS among men who have sex with men. Age-based inequalities also found in terms of incorrect diagnosis |
| **Heaphy and Yip (2003)** ***data draws from the same studies but treated differently due to differing emerging themes** | Considers how non-heterosexual ways of living differ in terms of identity, relationships and community. | Mixed methods Data are taken from six initial focus groups (three with women, three with men) with self-identified lesbians and gay men. As part of a programme of research further focus groups and a survey were also conducted and are reported as separate studies below. | Self-identified lesbians and gay men. | Aged 50+ | Loneliness/social isolation Access to social care Violence | Narrative connecting analysis undertaken exploring processes and understandings of how being LGBT shapes relationships. The authors were careful not to oversimplify relationships as being indicative of inequality. | Some evidence of sexuality-based and age-based health inequalities. The importance and difficulties in creating LGBT social networks are discussed. Homophobia and violence also shape health inequalities experienced by older LGBT people. |
| **Heaphy, Yip and Thompson (2004)** Heaphy (2007) Heaphy, Yip and Thompson (2003) ***data draws from the same studies but treated differently due to differing emerging themes** | Examines the difference that being non-heterosexual makes to how people experience ageing and later life. *Note, different themes and data presented to the study above | Mixed methods Data are taken from six initial focus groups (three with women, three with men), and from participants who completed a questionnaire. A sub-sample of 20 participants were later interviewed. Study presents results from the questionnaire, focus groups and interviews. | Gay men, Bisexual men, Gay and lesbian women, bisexual women | In the quantitative sample, among men (n=66 aged 50-59 years; n=56 60-69 years; n=42 aged 70+); among women (n=79 aged 50-59 years; n=20 60-69 years; n=3 aged 70+); women not well representative in the oldest age groups | Loneliness/social isolation Access to social care Violence | The presented narrative connecting analysis, using focus group and interview data to explore trends in the quantitative evidence of the link between sexuality and health and care outcomes. The paper also present discrepant cases to emphasise the diversity of experience. | Some evidence of sexuality-based inequalities is presented, with some LGBT individuals feeling disempowered particularly in anticipation of formal care settings. However, the authors describe a mixed picture, with no one narrative prevailing. |
| **(Heaphy 2009)** ***data draws from the same studies but treated differently due to differing emerging themes** | Examines how gay men and lesbians over 50 years old structure and negotiate their relational lives and the factors that limit negotiation *Note, different themes and data presented to the study above | Mixed methods Data are taken from six initial focus groups (three with women, three with men), and from participants who completed a questionnaire. A sub-sample of 20 participants were later interviewed. Study presents results from the questionnaire, focus groups and interviews. | Gay men, Bisexual men, Gay and lesbian women, bisexual women | In the quantitative sample, among men (n=66 aged 50-59 years; n=56 60-69 years; n=42 aged 70+); among women (n=79 aged 50-59 years; n=20 60-69 years; n=3 aged 70+); women not well representative in the oldest age groups | Mental health Loneliness/social isolation | Narrative connecting analysis is presented that forms connections between sexuality and risk or actual mental health issues | Implicit connections are made between sexuality and loneliness and social isolation, with the authors presenting evidence where limited economic resources combine with diminished social resources to limit intimate and relational possibilities for some older LGBT people, which can lead to self-concealment and feelings of loneliness and isolation. As with the papers authored by Heaphy above, a nuanced and complex picture is formed of how this manifests. |
| **Hubbard and Rossington (1995)** | Primarily explores the housing need and preferences of older LGBT people, includes evidence on health and care experiences | Mixed methods: survey and series of interviews | Gay men and lesbian women | N=31 women and N=59 men were included in the survey | Access to healthcare Violence | Narrative connections made between sexuality and poorer health outcomes | Sexuality-based inequalities are implicit for some outcomes and explicit for others. |
| **Ingham, et al. (2017)** | Explores experiences of same-sex partner bereavement in women over the age of 60. | Qualitative Semi-structured interviews | Gay or Lesbian women (none self-defined as bisexual) | N=8 women participated aged 61-74 yrs with a mean age of 68.3 years | Loneliness/social isolation Access to end of life care | Narrative connections made between experiences of homophobia during bereavement | Sexuality-based inequalities apparent during the bereavement process as women describe rejection from partners’ families and unfair treatment from care providers. |
| **Jones, et al. (2013)** Fenge and Jones (2011) | Action research project aimed at empowering older lesbians and gay men in rural areas | Qualitative Biographic Narrative Interviews, focus group and ethnographic methods | Gay men Gay or Lesbian women | All participants were aged 55 and over; biographic interviews (n=7), focus group (n=12) and filming and interviews (n=3). | Mental health | Sexuality based inequalities identified through intensive processual involvement with biographies and their impact on ageing explored through a variety of qualitative methods. | Sexuality-based inequalities apparent including those related to suicide and suicidal ideation in participant’s accounts, which were directly related to their sexuality. |
| **King and Stoneman (2017)** | Study explored older LGBT people’s housing concerns, preferences and experiences. | Mixed methods: study involving focus groups and online questionnaire | Gay men, Bisexual men, Gay or Lesbian women, Bisexual women, pansexuals, trans-identifying | All aged 50+ (n=26 for focus groups and n=191 for survey). Gender identity in survey: 7% trans; sexual identity in survey: Lesbian/ gay women (42%), Gay men (43%), Bisexual women (5%); Bisexual men (2%); Other (4%) | Loneliness/social isolation Access to social care Violence | Narrative connecting analysis linking sexuality with health and social care providers with aspirations and fears for future care | Participants expressed concern about the ability of social networks to provide informal social care, as well as the extent and quality of social care for sexual minorities. Sexuality-related fears about safety in housing were also evidenced. |
| **Phillips and Knocker (2010)** Knocker, et al. (2012) | Evaluation report of the Opening Doors project, an LGBT social group for older people in London and SE England | Mixed methods: routine data and interviews | Gay men, Gay or Lesbian women | All members of group aged 50+ | Loneliness/social isolation | Counterfactual reasoning and narrative connecting analysis | Participants reflected on the importance of an older LGBT group in their lives, the impact that the presence of such a group made, and the way in which social networks had been developed and the way in which the group had contributed to staving off feelings of loneliness. |
| **Langley (2001)** | Examined how older lesbian women and gay men perceived their needs should they become ill or disabled as they age | Mixed methods: A questionnaire was used and 8/19 respondents participated in in-depth interviews | Gay men, Gay or Lesbian women (none appeared to self-identify as bisexual) | Interview participants aged between 51 and 68 (n=4 men and n=4 women). | Access to healthcare Loneliness/social isolation Access to social care | Narrative connecting analysis linking past experiences with health and social care providers with aspirations and fears for future care | All participants expressed concerns mirroring those of non-LGBT people around ageing and loss of independence. Sexuality-based inequalities were apparent in the responses through fear of homophobia in care settings. |
| **Keogh, et al. (2004)** | Explores healthcare usage among gay men | Mixed methods: large survey and small number of interviews | Gay men | Gay men of all ages; over 50s disaggregated | Access to health care | Theoretical link made between sexuality and health care access | Sexuality-based inequality based on concealing identity. Narrow scope in relevance for review. |
| **Lawrence and Cross (2013)** | Examines experience of the first ageing HIV cohort | Qualitative: semi-structured interviews | Gay men | N=7 gay men aged 40-70 years with a median age of 55. | Mental health Access to social care HIV/AIDS Violence | Narrative connecting analysis presented linking diagnosis of HIV as a trigger for stigma | Sexuality-based inequalities are implicit with the higher risk of contracting HIV/AIDS among men who have sex with men. HIV diagnosis was trigger for further stigma; this stigma was expected to increase with age. |
| **McParland and Camic (2018)** | Explored what it means to experience dementia among older LGBT people and their carers. | Qualitative Semi-structured interviews. | Gay men; Gay and Lesbian women; LGBT carers (note bisexuality not mentioned) | 10 interviews conducted; mean age of people with dementia was 74.1 years (range 57–83) and the mean age of significant others was 69.3 years (range 43–83). | Access to social care | Narrative connecting analysis of processes and events distinguishing between challenges commonly experienced by those with a dementia diagnosis and those seemingly distinct to lesbian and gay individuals. | Sexuality-based inequalities were manifest, sometimes described as a ‘double stigma’ arising from living with dementia and from being lesbian or gay. |
| **Owen and Catalan (2012)** | An exploration of the lived experience of ageing in gay men. | Qualitative biographical narrative interviews | Gay men; Bisexual men (specifically HIV +ve) | Ten interviews with men aged 52-78 years | Loneliness/social isolation Access to social care HIV/AIDS | Evidence of sexuality-based inequalities based on intensive processual involvement from biographical narrative interviews | Sexuality-based inequalities uncovered as older HIV+ve men age and become socially isolated |
| **Parslow and Hegarty (2013)** | Explore the experiences of lesbian women with family elder care responsibilities. | Qualitative Interviews | Gay or Lesbian women (bisexual women not mentioned) | N=6 women interviewed aged 48-62 years | Access to social care | Narrative connecting analysis presents counterevidence to prevailing heteronormative understandings of the care experience | Sexuality-based inequalities in the caring experience uncovered with women becoming involved in caring having their lesbian identities come under threat and re-positioned as heterosexual by default |
| **Patel, et al. (2016)** | Measured the prevalence of non-infectious illnesses and their risk factors and described healthcare use in among HIV positive patients aged 50+ | Quantitative Survey of clinic attendees who were HIV+ve | Gay and bisexual men: 87% (n=260) of survey respondents were men who have sex with men. | N=260 gay and bisexual men, all clinic attendees and all aged 50+ | HIV/AIDS Drug use Smoking Exercise Alcohol Access to healthcare Long-term illness | Theoretical or mechanistic evidence presented to support high rates of comorbidity among older people with HIV, with risk factors including increasing age, HIV and ART. | Sexuality-based inequalities are implicit with the higher risk of contracting HIV/AIDS among men who have sex with men. Chronic conditions commonly found in older individuals in the general population are highly prevalent among HIV-positive individuals aged 50+. |
| **Piatczanyn, Bennett and Soulsby (2016)** | Explore whether gay widowers face the same challenges and whether there are additional challenges compared to heterosexual widowers following the loss of their partners. | Qualitative Interviews | Gay men who were widowers | Mainly older men (n=20): Mean 56.2 (range: 37–83) | Loneliness/social isolation Mental health Access to end of life care | Narrative connecting analysis is presented that outlines sexuality-based dis-enfranchisement in the bereavement experiences of gay men. | Sexuality-based inequalities manifest in experiences of loneliness, end of life care and mental health resulting from dis-enfranchisement, especially of grief, based on being gay. |
| **Price (2010)** ****data draws from the same studies but treated differently due to differing emerging themes** | Explore how sexuality impacts upon providing dementia care | Qualitative Semi-structured interviews | Gay men, Gay or Lesbian women | Dementia carers ranged in age (20-69), but over half were aged >50 | Access to social care | Narrative connecting analysis explored experiences and made connections between dementia carer experiences and sexuality | Sexuality-based inequalities manifest ranging from ambivalence through to hostility and explicit homophobia in access to care and treatment of LGBT informal carers. |
| **Price (2012)** ****data draws from the same studies but treated differently due to differing emerging themes** | Explore how sexuality and caring experiences shapes carers’ own care plans and aspirations | Qualitative Semi-structured interviews | Gay men, Gay or Lesbian women | Dementia carers ranged in age (20-69), but over half were aged >50 | Loneliness/social isolation Access to social care | Narrative connecting analysis explored the extent to which carer perceptions are grounded in reality | Sexuality-based inequalities manifest as LGBT carers’ accounts express sense of fear and anxiety about a future which, would not be the same for heterosexual people. |
| **Simpson (2013b)** (Simpson 2012) (Simpson 2013a) (Simpson 2014) | Explore middle-aged gay men’s accounts of friendship. | Qualitative In-depth interviews | Gay men (bisexual men not mentioned) | N=27 men; aged 39-61 years | Loneliness/social isolation Violence | Narrative connecting analysis describing importance of friendship family in traversing social structures and discourses, including (gay) ageism, that impose limits on self-expression/relating. | Sexuality-based inequality manifest as social isolation in gay men’s accounts of needing to live a double life on occasion; describe increased threat of violence based on sexuality. |
| **Simpson (2016)** | Explored middle-aged gay men’s accounts of Manchester’s gay voluntary organizations | Qualitative In-depth interviews | Gay men who were regular attendees or volunteers at gay voluntary organisations | Defined as middle-aged (39-55 years; n=20) | Loneliness/social isolation | Narrative connecting analysis explored accounts of how men were involved with voluntary organisations and the impact of involvement | Some evidence of sexuality-based inequality but more precisely around the important role of voluntary organisations as meeting spaces for older gay men and offsetting the potential risk of social isolation and loneliness. |
| **Smith (1992)** | This article presents a psychotherapist  perspective demonstrating that therapy can be helpful and relevant to older lesbians when the patient's lesbianism is not construed as a basic (psychiatric) problem | Qualitative: case series | Lesbian women | Six case studies are included | Access to social care Loneliness/social isolation Mental health | Narrative connecting analysis provided illuminating link between negative experiences of social care and health care and sexuality | This study explores a link between unequal treatment from health and care providers and patients’ sexuality; it provides examples of where loneliness, isolation and unequal treatment have contributed to poorer mental health outcomes that reversed, improved or stabilized with correct treatment. |
| **Traies (2015)** | This article explores the nature of friendship and community among old lesbians | Quantitative: Survey. | Gay or Lesbian women; Doesn't explicitly mention bisexual identity | N=372 women aged 60 to 90 of whom 45 were aged 70 or over, and 9 were over 80. | Loneliness/social isolation Mental health | None provided explicitly, although implicit discussion that maintaining friendships and networks more challenging because of sexuality | This study considered the role of friendship as a protective factor against loneliness and social isolation and promotion of mental health |
| **Traies (2016)** | This book aims to shed light on older Lesbian women, a population considered neglected within the LGBT acronym | Mixed methods: Survey and in-depth interviews | Lesbian women; Doesn't explicitly mention bisexual identity | 400 women born 1910-1950 aged 60 to 90 participated in the survey. 45 women were interviewed in-depth  (aged 60-91) | Access to healthcare; Access to social care | Narrative connecting analysis provided illuminating link between negative experiences of social care and health care and sexuality | This study provided comprehensive evidence on the lives and health and social care experiences of lesbian women in the UK, and provided evidence of the way in which hostile experiences could be traced to disclosure of sexual identity (either voluntary or involuntary disclosure) |
| **Ward, River and Fenge (2008)** | Interventions aimed at understanding and enhancing provision for older people and their carers. | Mixed methods: Action research | Gay Men, Gay or Lesbian women; Bisexual not stated | Volunteers were self-identified lesbian and gay men, ranging in age from 55 to 90 years (Initially n=40). | Access to social care | Evidence of reversibility; initially, common to both projects were frequent encounters with service providers and policy makers who had employed terms such as “hard to reach”, “hidden,” and “invisible,” to collectively label older lesbians and gay men. However, both initiatives demonstrated that older lesbian and gay people are often silenced by policy and practice which actively excludes and marginalises their needs and perspectives. | Evidence of sexuality-based inequalities in access to health and social care: Prior to the projects no systematic attempts had been made to gather information on older members of the lesbian and gay communities or to develop services tailored to their needs. Health and local authority representatives approached by the projects repeatedly questioned the existence of LGBT in their locality or claimed their low numbers did not warrant work in this area |
| **Warner, et al. (2003)** | Exploring the psychological health and quality of life of older gay men and lesbians in the UK. | Quantitative Survey | Gay men, gay and lesbian women *bisexual not mentioned | N=85 gay men; n=26 lesbians; n=14 heterosexuals. Mean age of the gay/lesbian respondents was 65 (SD 6.4, range 56–81) | Mental health | High levels of common mental health disorders were found, although these were not directly linked with sexuality. | High levels of psychological distress/morbidity with 16% of respondents scoring above the threshold on the GHQ-28. |
| **Westwood (2016)** *****data draws from the same studies but treated differently due to differing emerging themes** | Explores the lack of choice in sheltered housing and residential/nursing care provision for older lesbian, gay and bisexual (LGB) individuals in the UK. | Qualitative Semi-structured interviews. | Gay men, Bisexual men, Gay or Lesbian women, Bisexual women | N=60 participants (n=36 women and n=24 men); Ages ranged from 58-92 years for women and 58-76 years for men; the mean age was 64 for both. | Access to social care | Narrative connecting analysis presented highlighting the need both to address heteronormativity in older age provision, and lack of choice in housing/care options. | Sexuality-based inequalities manifest in older LGB individuals’ concerns about mainstream sheltered accommodation and residential care including lack of visibility, risky visibility, unequal openness and compulsory co-occupation of care spaces. |
| **Westwood (2017a)** *****data draws from the same studies but treated differently due to differing emerging themes** | Explores the ‘right to die’ debate from the perspectives of older lesbians and gay men | Qualitative Semi-structured interviews. | Gay men, Bisexual men, Gay or Lesbian women, Bisexual women | N=60 participants (n=36 women and n=24 men); Ages ranged from 58-92 years for women and 58-76 years for men; the mean age was 64 for both. | Loneliness/social isolation Access to social care | Narrative connecting analysis presented making connections between (anticipated) social care and end of life care events and status of being lesbian, gay or bisexual | Sexuality-based inequalities manifest as some participants understood their sexualities as informing their relative lack of informal social support and in particular intergenerational support, which in turn increased their risk of exposure to formal services which would not meet their needs. |
| **Westwood (2017b)** *****data draws from the same studies but treated differently due to differing emerging themes** | Explores how older lesbian, gay, and bisexual (LGB) people in the United Kingdom engage with religion in later life. Particular consideration was given to the equality implications, in the form of parity of participation, including in the context of religious-based social care provision. | Qualitative Semi-structured interviews. | Gay men, Bisexual men, Gay or Lesbian women, Bisexual women | N=60 participants (n=36 women and n=24 men); Ages ranged from 58-92 years for women and 58-76 years for men; the mean age was 64 for both. | Access to social care | Narrative connecting analysis highlight the temporality of religious in/exclusions, where exclusionary narratives predominated, with some participants describing their first experiences of religious-based rejection in later life. | Sexuality-based inequalities manifest as some participants experienced homophobia and discrimination on new grounds in social care settings |
| **Wilkens (2015)** | Explores loneliness and isolation in older lesbians, looking at the benefits offered by a  over-55 lesbian social group in Yorkshire. | Qualitative Interviews and a focus group | Gay or Lesbian women | N=10 interviews with women with an average age of 62. | Access to healthcare Loneliness/social isolation | Narrative connecting analysis presented processes in life as trajectories of loneliness and resilience | Sexuality-based inequality implicit as many of the women, who were lonely or feared future loneliness, derived a sense of belongingness from the group specifically catered to lesbian women |
| **Wilkens (2016)** | Explores the impact of belonging to a same-sexuality social group or network for older lesbians and bisexual women, with a focus on isolation and loneliness | Qualitative Interviews | Gay or Lesbian women | N=35 interviews with women ranging in age from 57-73 years | Loneliness/social isolation | Narrative connecting analysis presented processes in life as trajectories of loneliness and resilience | Sexuality-based inequalities were implicit as the women included in the study discussed the difficulties of accessing mixed sexuality spaces and the feelings of loneliness and isolation that a lack of access to lesbian social groups could bring forth. |
| **Willis, et al. (2016)** | Examines the ways in which older people’s residential and nursing homes can constitute heteronormative environments | Qualitative Interviews and focus group | Gay men, bisexual men, Gay or Lesbian women, bisexual women | 5 focus groups;  n=29 interviews; all aged 50-76 years. | Access to social care | Narrative connecting analysis offered looking at narratives of perception | Sexuality-based inequalities manifest as care spaces regarded as hetero-sexualised spaces in which the discussion and expression of non-heterosexual identities and sexual practices is absent |

# References

Almack, K., Seymour, J. and Bellamy, G. 2010. Exploring the impact of sexual orientation on experiences and concerns about end of life care and on bereavement for lesbian, gay and bisexual older people. *Sociology*, **44**, 5, 908-924.

Bonell, C.P., Hickson, F.C., Weatherburn, P. and Reid, D.S. 2010. Methamphetamine use among gay men across the UK. *International journal of drug policy*, **21**, 3, 244-246.

Bouman, W.P., Claes, L., Marshall, E., Pinner, G.T., Longworth, J., Maddox, V., Witcomb, G., Jimenez-Murcia, S., Fernandez-Aranda, F. and Arcelus, J. 2016. Sociodemographic variables, clinical features, and the role of preassessment cross-sex hormones in older trans people. *The journal of sexual medicine*, **13**, 4, 711-719.

Clover, D. 2006. Overcoming barriers for older gay men in the use of health services: a qualitative study of growing older, sexuality and health. *Health Education Journal*, **65**, 1, 41-52.

Colledge, L., Hickson, F., Reid, D. and Weatherburn, P. 2015. Poorer mental health in UK bisexual women than lesbians: evidence from the UK 2007 Stonewall Women's Health Survey. *Journal of Public Health*, **37**, 3, 427-437.

Cronin, A. and King, A. 2014. Only connect? Older lesbian, gay and bisexual (LGB) adults and social capital. *Ageing and Society*, **34**, 2, 258-279.

Dickinson, T., Cook, M., Playle, J. and Hallett, C. 2012. 'Queer' treatments: Giving a voice to former patients who received treatments for their 'sexual deviations'. *Journal of Clinical Nursing*, **21**, 9-10, 1345-1354.

Elford, J., Ibrahim, F., Bukutu, C. and Anderson, J. 2008. Over fifty and living with HIV in London. *Sexually Transmitted Infections*, **84**, 6, 468-472.

Fenge, L.-A. 2008. Striving towards inclusive research: An example of participatory action research with older lesbians and gay men. *British Journal of Social Work*, **40**, 3, 878-894.

Fenge, L.-A. and Fannin, A. 2009. Sexuality and bereavement: Implications for practice with older lesbians and gay men. *Practice: Social work in action*, **21**, 1, 35-46.

Fenge, L.-A. and Jones, K. 2011. Gay and pleasant land? Exploring sexuality, ageing and rurality in a multi-method, performative project. *British Journal of Social Work*, **42**, 2, 300-317.

Guasp, A. 2011. *Lesbian, Gay and Bisexual People later in life*. Stonewall, London.

Hargreaves, M., Fuller, G. and Gazzard, B. 1988. Occult AIDS: pneumocystis carinii in elderly people. *BMJ*, **297**, 6650, 721.

Heaphy, B. 2007. Sexualities, gender and ageing: Resources and social change. *Current Sociology*, **55**, 2, 193-210.

Heaphy, B. 2009. Choice and its limits in older lesbian and gay narratives of relational life. *Journal of GLBT Family Studies*, **5**, 1-2, 119-138.

Heaphy, B., Yip, A. and Thompson, D. 2003. The social and policy implications of non-heterosexual ageing-Selective findings. *Quality in Ageing and Older Adults*, **4**, 3, 30-35.

Heaphy, B. and Yip, A.K. 2003. Uneven possibilities: Understanding non-heterosexual ageing and the implications of social change. *Sociological research online*, **8**, 4, 1-12.

Heaphy, B., Yip, A.K. and Thompson, D. 2004. Ageing in a non-heterosexual context. *Ageing & Society*, **24**, 6, 881-902.

Hubbard, R. and Rossington, J. 1995. *As we grow older: A study of the housing and support needs of older lesbians and gay men*. Polari Housing Association, London.

Ingham, C.F., Eccles, F.J., Armitage, J.R. and Murray, C.D. 2017. Same-sex partner bereavement in older women: an interpretative phenomenological analysis. *Aging & mental health*, **21**, 9, 917-925.

Jones, K., Fenge, L.-A., Read, R. and Cash, M. 2013. Collecting Older Lesbians' and Gay Men's Stories of Rural Life in South West England and Wales. In  *Forum: Qualitative Social Research*. Bournemouth University, Fern Barrow, Poole, Dorset, BH12 5BB, UK.

Keogh, P., Weatherburn, P., Henderson, L., Reid, D., Dodds, C. and Hickson, F. 2004. *Doctoring gay men: exploring the contribution of General Practice*. Sigma Research, London.

King, A. and Stoneman, P. 2017. Understanding SAFE Housing–putting older LGBT* people’s concerns, preferences and experiences of housing in England in a sociological context. *Housing, Care and Support*, **20**, 3, 89-99.

Knocker, S., Maxwell, N., Phillips, M. and Halls, S. 2012. Opening doors and opening minds. *Lesbian, Gay, Bisexual and Transgender Ageing: Providing Effective Support Through Biographical Practice, Jessica Kingsley Publishers, London and Philadelphia, PA*, 150-64.

Langley, J. 2001. Developing anti‐oppressive empowering social work practice with older lesbian women and gay men. *British Journal of Social Work*, **31**, 6, 917-932.

Lawrence, S. and Cross, M. 2013. Life transition with HIV: Some observations of the phenomenon of growing older with the infection. *Counselling Psychology Review*, **28**, 1, 25-36.

McParland, J. and Camic, P.M. 2018. How do lesbian and gay people experience dementia? *Dementia*, **17**, 4, 452-477.

Owen, G. and Catalan, J. 2012. ‘We never expected this to happen’: narratives of ageing with HIV among gay men living in London, UK. *Culture, health & sexuality*, **14**, 1, 59-72.

Parslow, O. and Hegarty, P. 2013. Who cares? UK lesbian caregivers in a heterosexual world. In  *Women's Studies International Forum*. Elsevier, 78-86.

Patel, R., Moore, T., Cooper, V., McArdle, C., Perry, N., Cheek, E., Gainsborough, N. and Fisher, M. 2016. An observational study of comorbidity and healthcare utilisation among HIV-positive patients aged 50 years and over. *International journal of STD & AIDS*, **27**, 8, 628-637.

Phillips, M. and Knocker, S. 2010. Opening Doors Evaluation: The Story So Far. In  *Opening Doors* Age UK, London.

Piatczanyn, S.A., Bennett, K.M. and Soulsby, L.K. 2016. “We Were in a Partnership That Wasn’t Recognized by Anyone Else” Examining the Effects of Male Gay Partner Bereavement, Masculinity, and Identity. *Men and Masculinities*, **19**, 2, 167-191.

Price, E. 2010. Coming out to care: gay and lesbian carers’ experiences of dementia services. *Health & Social Care in the Community*, **18**, 2, 160-168.

Price, E. 2012. Gay and lesbian carers: Ageing in the shadow of dementia. *Ageing & Society*, **32**, 3, 516-532.

Simpson, P. 2012. Perils, precariousness and pleasures: Middle-aged gay men negotiating urban ‘heterospaces’. *Sociological Research Online*, **17**, 3, 1-10.

Simpson, P. 2013a. Alienation, ambivalence, agency: Middle-aged gay men and ageism in Manchester’s gay village. *Sexualities*, **16**, 3-4, 283-299.

Simpson, P. 2013b. Differentiating the self: The kinship practices of middle-aged gay men in Manchester. *Families, Relationships and Societies*, **2**, 1, 97-113.

Simpson, P. 2014. Differentiating selves: middle‐aged gay men in Manchester's less visible ‘homospaces’. *The British journal of sociology*, **65**, 1, 150-169.

Simpson, P. 2016. The resources of ageing? Middle‐aged gay men's accounts of Manchester's gay voluntary organizations. *The Sociological Review*, **64**, 2, 366-383.

Smith, P.P. 1992. Encounters with older lesbians in psychiatric practice. *Sexual and marital therapy*, **7**, 1, 79-86.

Traies, J. 2015. Old lesbians in the UK: Community and friendship. *Journal of lesbian studies*, **19**, 1, 35-49.

Traies, J. 2016. *The Lives of Older Lesbians: Sexuality, Identity & the Life Course*. Springer.

Ward, R., River, L. and Fenge, L.-A. 2008. Neither silent nor invisible: a comparison of two participative projects involving older lesbians and gay men in the United Kingdom. *Journal of Gay & Lesbian Social Services*, **20**, 1-2, 147-165.

Warner, J.P., Wright, L., Blanchard, M. and King, M. 2003. The psychological health and quality of life of older lesbians and gay men: A snowball sampling pilot survey. *International Journal of Geriatric Psychiatry*, **18**, 8, 754-755.

Westwood, S. 2016. ‘We see it as being heterosexualised, being put into a care home’: gender, sexuality and housing/care preferences among older LGB individuals in the UK. *Health & social care in the community*, **24**, 6, e155-e163.

Westwood, S. 2017a. Older Lesbians, Gay Men and the ‘Right to Die’Debate: ‘I Always Keep a Lethal Dose of Something, Because I don’t Want to Become an Elderly Isolated Person’. *Social & Legal Studies*, **26**, 5, 606-628.

Westwood, S. 2017b. Religion, sexuality, and (in) equality in the lives of older lesbian, gay, and bisexual people in the United Kingdom. *Journal of Religion, Spirituality & Aging*, **29**, 1, 47-69.

Wilkens, J. 2015. Loneliness and belongingness in older lesbians: The role of social groups as “community”. *Journal of Lesbian Studies*, **19**, 1, 90-101.

Wilkens, J. 2016. The significance of affinity groups and safe spaces for older lesbians and bisexual women: creating support networks and resisting heteronormativity in older age. *Quality in Ageing and Older Adults*, **17**, 1, 26-35.

Willis, P., Maegusuku-Hewett, T., Raithby, M. and Miles, P. 2016. Swimming upstream: the provision of inclusive care to older lesbian, gay and bisexual (LGB) adults in residential and nursing environments in Wales. *Ageing & Society*, **36**, 2, 282-306.
